# *Escherichia coli* associated hematogenous sternoclavicular joint osteomyelitis: A rare condition with a rare causative pathogen

**DOI:** 10.1016/j.idcr.2022.e01381

**Published:** 2022-01-06

**Authors:** Tyler Luu, Gail Reid, Brian Lavery

**Affiliations:** a Loyola University Medical Center, Department of Internal Medicine, 2160 S 1st Avenue, Maywood, IL 60153, USA; b Edward Hines, Jr. VA Hospital, Department of Internal Medicine, 5000 5th Ave, Hines, IL 60141, USA

**Keywords:** *E. coli*, Prostatic abscess, UTI, Osteomyelitis, Sternoclavicular joint osteomyelitis

## Abstract

*Escherichia coli* is the most common microorganism that causes urinary tract infections (UTIs), including acute prostatitis. However, *E. coli* osteomyelitis, especially ones that involve the sternoclavicular joint, are rare hematogenous complications. We present a case of an immunocompetent man who presented with symptoms of UTI and right shoulder pain. Urine cultures and blood cultures grew *E. coli*. There was radiographic evidence of prostatic abscess and a right sternoclavicular joint osteomyelitis. This case is unique given the rare occurrence of non-traumatic clavicular bacterial osteomyelitis and the type of bacteria involved. In conclusion, it is important for clinicians to be aware of *E. coli* sternoclavicular osteomyelitis in adults with preceding bacterial prostatitis.

## Introduction

*E. coli* is the most common microorganism that causes urinary tract infections (UTIs) [1]. It can also cause osteomyelitis, a bone infection. Hematogenous osteomyelitis, a subtype of osteomyelitis, is bone infection that has been seeded through the bloodstream. The sternoclavicular joint (SCJ) is where the clavicle attaches to the manubrium remains one of the rare locations for hematogenous osteomyelitis, with approximately less than 250 cases reported in the past 50 years [2]. In this case study, we present a case of SCJ osteomyelitis as a complication of *E. coli* prostatitis, which we had not found reported in literature.

## Case

**Fig. 1:**
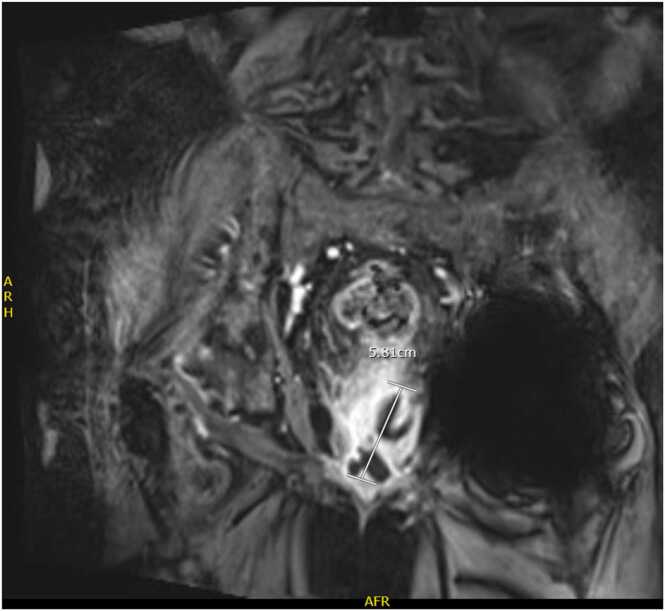
MRI of the abdomen and pelvis shows a demonstration of a 3.9 × 2.8 × 5.8 cm collection with irregular, thick enhancing walls arisen from the left peripheral zone mid base of the prostate gland.

**Fig. 2:**
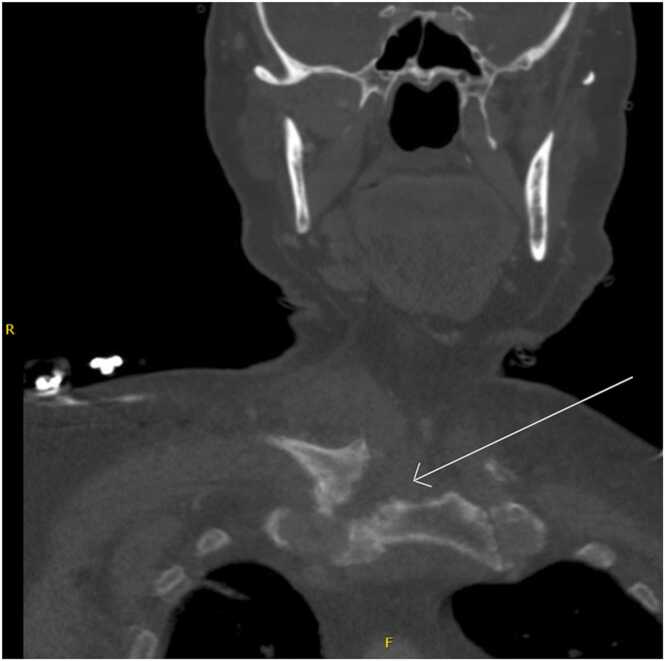
White arrow shows the progressive erosive changes at the right sternoclavicular joint with erosions involving the distal clavicle and adjacent sternum, consistent with acute osteomyelitis of the sternoclavicular joint.

We have an 81-year-old male veteran with past medical history of atrial fibrillation on chronic anticoagulation with Apixaban, heart failure with reduced ejection fraction, hypertension, non-obstructed coronary artery disease, chronic non-occluding DVT of L gastric vein, stage IIB non-squamous cell lung carcinoma s/p resection, and chronic poly-osteoarthritis most prominently of the bilateral hip joints secondary to osteoarthritis, who presented to the emergency department with a one week duration of progressively worsening right shoulder pain, associated with fevers and chills. Patient denied any preceding injury to the right shoulder. Pain was localized and achy in nature, associated with markedly reduced range of motion and was different from his usual arthritis. Our patient also endorsed a 2-week history of urinary symptoms including increased urinary frequency, draining with urination, dysuria as well as suprapubic tenderness. He denied any flank pain back pain. On admission, patient was febrile to 101°F. Laboratory work-up revealed leukocytosis and elevated inflammatory markers. Urine cultures and blood cultures were positive for *E. coli* that was susceptible to all tested antimicrobials. CT and MRI of the abdomen and pelvis demonstrated a large multi-septated thick walled collection, arising from the prostate that was consistent with an acute prostatic abscess formation (Fig. 1). Due to patient's complaint of shoulder pain, serial imaging of right shoulder was performed. We first we obtained a plain film of the right shoulder which was negative for acute fracture or dislocation. Subsequently CT head and neck with IV contrast revealed progressive erosive changes at the right sternoclavicular joint, compatible with osteomyelitis (Fig. 2). Transthoracic echocardiogram was obtained without evidence of infective endocarditis.

## Decision-making

With blood culture positive for *E. coli*, together with consistent radiographic findings, our patient was diagnosed with *E. coli* bacteremia with right sternoclavicular joint osteomyelitis as a complication. Source of infection was believed to be urinary origin. Infectious disease was consulted and patient was treated with 6 weeks of intravenous Ampicillin-Sulbactam, followed by suppressive therapy with Sulfonamide-Trimethoprim due to his extensive infection. Patient improved significantly on antibiotic therapy with resolution of his UTI and right shoulder pain. We continued to assess patients’ improvement through repeat radiography. MRI abdomen pelvis 12 weeks later demonstrated resolution of prostatic abscess. At patient’s 4 month follow up visit with infectious disease, right shoulder pain completely resolved and his suppressive antibiotic was discontinued.

## Discussion

Hematogenous osteomyelitis is the results of bacteremic seeding of bone. In adults, it often involves the vertebrae, long bones, and pelvis [3]. Distal location such as the clavicles are an extremely rare site. Sternoclavicular joint is where the clavicle attaches to the manubrium. It remains a rare site of infection with approximately less than 250 cases reported in the past 50 years [2]. The sternoclavicular joint infection can occur via different pathways. One of them involves a direct inoculation of the joint or contiguous spread from a nearby area. The other pathway, which is more common is hematogenous spread via the bloodstream [4]. In terms of pathogenic organisms, *Staphylococcus aureus* is the most common [3]. Though there is no clear data on the most common causative agent of SCJ osteomyelitis, Ross Et all demonstrated that *Staph. aureus* was also the most common causative pathogen (49%), including *MSSA* and *MRSA*, followed by *P. aeruginosa* and *B. melitensis*. This was done by reviewing 180 patients with SCJ septic arthritis [5]. *E. coli* has been reported in a few hematogenous osteomyelitis cases, but none was associated with SCJ that we could find in literature.

*“Escherichia coli”* was named after Theodor Escherich, a German-Austrian pediatrician who first discovered this organism in the feces of healthy individuals [6]. *E. coli* is a Gram-negative, facultative anaerobic, rod-shaped, coliform bacterium of the genus Escherichia that is commonly found in the lower intestine of warm-blooded organisms, including human [7]. E. coli are enteric bacteria that in normal circumstances, positively add to the gut's regular function. Once E. coli reach tissues outside of the GI tract, they can become pathogenic and cause infections. Some of those common sites include the biliary tract, the urinary tract, as well as the intra-abdominal cavity [8]. The aforementioned sites have one thing in common is that they are in close proximity to the GI tract. Although rare, other extra intestinal infections include emphysematous pyomyositis, septic arthritis, spontaneous meningitis, and non-vertebral hematogenous osteomyelitis [9].

History and physical is important in assessing patientspresent with urinary symptoms and acute on chronic joint pain. Any bone pain that is localized in individuals with symptoms suggestive of systemic bacterial infections, including fevers, malaise, hypotension, and tachypnea should raise suspicion for hematogenous osteomyelitis [4]. In patients with history of polyarthritis, acute joint pain due to osteomyelitis could be easily overlooked and therefore missed diagnosed. Prompt diagnosis and appropriate treatment of SCJ osteomyelitis can avoid potential permanent functional impairment of the joint. They can also prevent further spread of infection into deeper tissues, including the adjacent posteriorly blood vessels, mediastinum, and pleural space which are rare but devastating consequences [10].

The diagnostic criteria of osteomyelitis in children and adults can be quite different. In children, acute osteomyelitis is often a clinical diagnosis. However, in adults, diagnosis requires both high clinical suspicion as well as subjective data including microbial cultures and radiographic studies [3]. When combined with convincing clinical and imaging evidence of infection, microbial cultures can obviate the need for additional invasive diagnostic testing such as a bone biopsy. Several imaging modalities have been used in the evaluation of suspected osteomyelitis. Cross-sectional imaging modalities like CT scanning and MRI are now considered standard in the diagnosis of osteomyelitis. Although expensive, they are sensitive and specific [11]. Broad spectrum antimicrobial therapy should be initiated promptly. Targeted therapy can be guided by culture susceptibility tests. It is worth noting that choosing an effective treatment for bacterial prostatitis is often challenging due to the poor prostatic tissue penetration of most antibiotics. In this case, Ampicillin/Sulbactam, a beta-lactam, achieves a lower level of penetration and should not be considered first drug of choice [12]. However due to patient’s significant clinical improvement on this regimen, decision was made to continue patient on it. Optimal duration of antibiotic treatment for acute bacterial prostatitis and acute uncomplicated hematogenous osteomyelitis is often 6 weeks. Length of therapy ultimately depends on patient’s clinical improvement. However, route of administrated can be quite different between acute prostatitis and osteomyelitis, with the former can be adequately treated with oral antibiotic while the latter often requires intravenous route for the best outcomes [3], [13].

## Conclusion

*E. coli* is a rare cause of SCJ osteomyelitis. Our patient is immunocompetent who does not have any risk factors for osteomyelitis. The history of urinary tract infection preceding any focal bone pain should alert clinicians of the possibility of bone involvement. Prompt diagnosis, and in turn, appropriate antibiotic treatment can result in successful patient outcomes and prevent devastated sequela.

## Ethical approval

This case report does not require review by ethics boardsAll of patient’s information has been protected. No names, initials or hospital MRN were used in this case report.

## Consent

Patient has been consented for this case reports.

## Funding

None.

## CRediT authorship contribution statement

Tyler Luu was directly involved in patient’s care, analyzed laboratory data, performed literature review and wrote the manuscript. Gail Reid edited the manuscript aided in literature review.Brian Lavery was directly involved in patient’s care, edited the manuscript and aided in literature review.

## Competing interests

The authors declare that they have no competing interests.
